# 2,2′-(*p*-Phenyl­ene)bis­(4,5-dihydro-1*H*-imidazol-3-ium) bis­(3-nitro­benzoate)

**DOI:** 10.1107/S1600536810053341

**Published:** 2011-01-08

**Authors:** Xiu-Mei Song, Jun-Jun Li, Xin-Hua Liu, Chun-Xia Ren, Shao-Ming Shang

**Affiliations:** a School of Chemical and Material Engineering, Jiangnan University, 1800 Lihu Road, Wuxi, Jiangsu Province 214122, People’s Republic of China; b College of Pharmacy, GuangDong Pharmaceutical University, Guangzhou, Guangdong Province 510006, People’s Republic of China

## Abstract

In the title compound, C_12_H_16_N_4_^+^·2C_7_H_4_NO_4_^−^, the complete 2,2′-(*p*-phenyl­ene)bis­(4,5-dihydro-1*H*-imidazol-3-ium) (bib) dication is generated by crystallographic inversion symmetry. The bib cations reside on crystallographic inversion centers, which coincide with the centroids of the respective benzene rings. In the cation, the imidazole ring adopts an envelop conformation with the flap atom displaced by 0.082 (3) Å from the plane through the other ring atoms. In the crystal, the cations and anions are linked through inter­molecular N—H⋯O hydrogen bonds, forming chains running along the *a* axis. C—H⋯O inter­actions also occur. Weak π–π contacts between the imidazole rings of bib and between the benzene rings of NB [centroid–centroid distances = 3.501 (1) and 3.281 (2) Å, respectively] may further stabilize the structure.

## Related literature

For general background to supra­molecular inter­actions, see: Jeffrey (1997 ▶). For the structures of metal complexes with imidazole ligands reported by our group, see: Ren, Ye, He *et al.* (2004 ▶); Ren, Ye, Zhu *et al.* (2004 ▶); Ren *et al.* (2007 ▶, 2009 ▶).
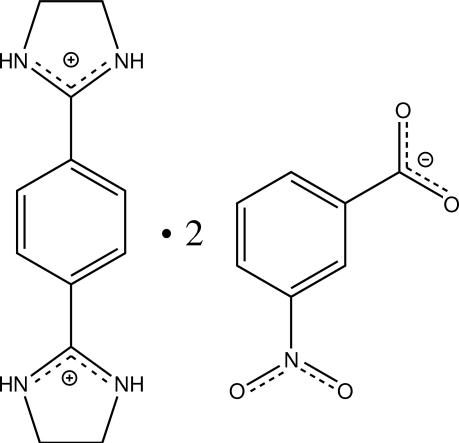


## Experimental

### 

#### Crystal data

C_12_H_16_N_4_^2+^·2C_7_H_4_NO_4_^−^
*M* *_r_* = 548.51
Triclinic, 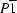

*a* = 6.9882 (12) Å
*b* = 7.4165 (12) Å
*c* = 13.233 (2) Å
α = 81.343 (3)°
β = 82.443 (3)°
γ = 62.699 (2)°
*V* = 600.96 (17) Å^3^
*Z* = 1
Mo *K*α radiation
μ = 0.12 mm^−1^
*T* = 273 K
0.67 × 0.55 × 0.42 mm

#### Data collection

Bruker SMART APEX CCD area-detector diffractometer
Absorption correction: multi-scan (*SADABS*; Bruker, 1998 ▶) *T*_min_ = 0.927, *T*_max_ = 0.953
3714 measured reflections
2575 independent reflections
1676 reflections with *I* > 2σ(*I*)
*R*_int_ = 0.016

#### Refinement

*R*[*F* ^2^ > 2σ(*F* ^2^)] = 0.053
*wR*(*F* ^2^) = 0.151
*S* = 1.07
2575 reflections
181 parameters
1 restraint
H-atom parameters constrained
Δρ_max_ = 0.20 e Å^−3^
Δρ_min_ = −0.28 e Å^−3^

### 

Data collection: *SMART* (Bruker, 1998 ▶); cell refinement: *SAINT-Plus* (Bruker, 1998 ▶); data reduction: *SAINT-Plus*; program(s) used to solve structure: *SHELXS97* (Sheldrick, 2008) ▶; program(s) used to refine structure: *SHELXL97* (Sheldrick, 2008) ▶; molecular graphics: *SHELXTL* (Sheldrick, 2008) ▶; software used to prepare material for publication: *SHELXTL*.

## Supplementary Material

Crystal structure: contains datablocks global, I. DOI: 10.1107/S1600536810053341/ds2078sup1.cif

Structure factors: contains datablocks I. DOI: 10.1107/S1600536810053341/ds2078Isup2.hkl

Additional supplementary materials:  crystallographic information; 3D view; checkCIF report

## Figures and Tables

**Table 1:** Hydrogen-bond geometry (Å, °)

*D*—H⋯*A*	*D*—H	H⋯*A*	*D*⋯*A*	*D*—H⋯*A*
C6—H6⋯O2^i^	0.93	2.48	3.283 (3)	144
N1—H1⋯O1^ii^	0.86	1.97	2.763 (3)	153
N2—H2⋯O2^iii^	0.86	1.80	2.646 (3)	166

Symmetry codes: (i) 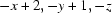; (ii) 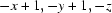; (iii) 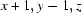.

**Fig. 1.**
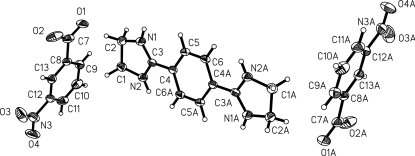
The structure of the title complex 1,4-bis(4,5-dihydro-H,4H-imidazol-2-yl)benzene bis(3-nitrobenzoate) showing 30° probability displacement ellipsoids.

**Fig. 2.**
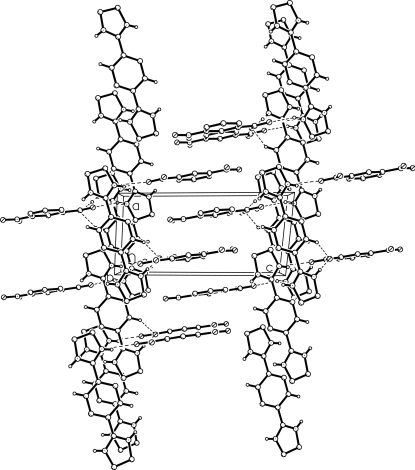
Crystal packing of the title compound viewed along the baxis. H atoms not involved in hydrogen bonding are omitted for clarity.

## supplementary crystallographic information

### Comment

Attention has been recently focused on the use of supramolecular interactions, such as hydrogen bonding and π-π interactions, in the controlled assembly of supramolecular architectures (Jeffrey, 1997). Hydrogen bonds often play a dominant role in crystal engineering because of their combine strength with directionality. We have reported several complexes having an imidazole entity, and have concluded that hydrogen bonding involving this group influences the geometry around the metal atom and the crystallization mechanism (Ren, Ye, He *et al.*, 2004; Ren, Ye, Zhu *et al.*, 2004; Ren, *et al.*, 2007; Ren, *et al.*, 2009). As a further contribution to this field, we describe herein the synthesis and crystal structure of the title compound.

The asymmetric unit of the title compound (Fig. 1) contains one-half of 1,4-bis(4,5-dihydro-*H*,4*H*–imidazol-2-yl)benzene (bib) cation and one 3-nitrobenzoate (NB) anion. In the bib molecule, the imidazole ring A (N1/N2/C1—C3) adopts an envelop conformation with atom C4 displaced by 0.082 (3)/A from the plane of the other ring atoms. Rings B (C4/C5/C6/C4'/C5'/C6') [symmetry code ('): 3 - *x*,-*y*,-*z*] are, of course, planar and they are oriented at a dihedral angle of 96.6/%.

In the crystal structure, the bib and NB ligands are joined together through hydrogen bonds between the carboxy oxygen in NB and nitrogen atom in bib to give a macrocycle N1—H1···O1 and N2—H2···O2 with the hydrogen bond geometry given in Table 1, and a face-to-face intracyclic π-π interaction at 3.50 (1) /A. Each bib group also features another macrocycles, resulting in 1-D chains running along the *a* axis. As illustracted in Fig. 2, the adjacent NB ligands are furthermore linked in the antiparallel alignment with offset along the *bc* plane by π-π contacts (3.28 (1)/A) in a 3-D structure (Fig. 2). Weak intermolecular C—H···O contacts contribute to the stability of the layered structure (Table 1).

### Experimental

All the reagents and solvents employed were commercially available and used as received without further purification.

Syntheses of 1,4-bis(4,5-dihydro-1*H*-imidazol-2-yl)benzene: a mixture of 1,4-Benzenedicarboxylic acid (2.31 g, 13.9 mmol), ethylenediamine (3.70 ml, 50 mmol), ethylenediamine dihydrochloride(6.64 g, 50 mmol) and toluene-*p*-sulfonic acid (0.208 g, 1.09 mmol) in ethyleneglycol (20 ml) was refluxed at 198/%C for 3 h. Then about half of the ethylene glycol solvent was then slowly removed by distillation at 120 /%C. The residue was dissolved in a mixture of water (40 ml) and concentrated hydrochloric acid (11 *M*, 3 ml). The addition of 50% aqueous sodium hydroxide gave a yellow precipitate that was recrystallized by methonal (yield 83% based on 1,4-benzenedicarboxylic acid (*ca* 2.50 g). Calc. for C_12_H_14_N_4_: C 67.27; H 6.59; N 26.15%. Found: C 66.98; H 6.92; N 26.08%. IR (KBr, cm^-1^): 3188(*m*), 2936(*m*), 2866(*m*), 1606(*s*), 1532(*s*), 1466(*s*), 1345(*m*), 1270(*s*), 1191(w), 1080(w), 981(*m*), 855(*m*).

Syntheses of the title compound: to a solution of 1,4-bis(4,5-dihydro-1*H*-imidazol-2-yl)benzene (0.0109 g, 0.05 mmol) in methonal (1 ml), an acetonitrile solution (1 ml) of 3-nitrobenzoic acid (0.0069 g, 0.06 mmol) was added and stirred 10 min at room temperature. The solution was allowed at room temperature in 10 ml diethyl diether for 25 h by slow evaporation. Colorless prismatic crystals of the title compound were obtained, which were collected by filtration, washed with water and dried in vacuum desiccator over silica gel (0.0095 g, 41%). IR (KBr,cm^-1^): 3436(*s*), 3080(w), 2924(*m*), 1618(*s*), 1560(*m*), 1524(*s*), 1383(*s*), 1370(*s*), 1351(*s*), 1282(*m*), 720(*m*), 696(w).

### Refinement

Anisotropic thermal parameters were applied to all nonhydrogen atoms. The organic hydrogen atoms attached to C atoms and N atom were fixed geometrically and treated as riding with C—H = 0.93 Å (aromatic) or 0.97 Å (methylene) and N—H = 0.86 Å with *U*_iso_(H) = 1.2 *U*_eq_(C or N). Rigid bond restraint instruction DELU was applied to improve the anisotropic displacement parameters involving N3 and C12.

### Crystal data

**Table:**

C_12_H_16_N_4_^2+^·2C_7_H_4_NO_4_^−^	*V* = 600.96 (17) Å^3^
*M**_r_* = 548.51	*Z* = 1
Triclinic, *P*1	*F*(000) = 286
Hall symbol: -P 1	*D*_x_ = 1.516 Mg m^−^^3^
*a* = 6.9882 (12) Å	Mo *K*α radiation, λ = 0.71073 Å
*b* = 7.4165 (12) Å	µ = 0.12 mm^−^^1^
*c* = 13.233 (2) Å	*T* = 273 K
α = 81.343 (3)°	Block, colorless
β = 82.443 (3)°	0.67 × 0.55 × 0.42 mm
γ = 62.699 (2)°	

### Data collection

**Table:**

Bruker SMART APEX CCD area-detector diffractometer	2575 independent reflections
Radiation source: fine-focus sealed tube	1676 reflections with *I* > 2σ(*I*)
graphite	*R*_int_ = 0.016
φ and ω scans	θ_max_ = 27.0°, θ_min_ = 1.6°
Absorption correction: multi-scan (*SADABS*; Bruker, 1998)	*h* = −8→8
*T*_min_ = 0.927, *T*_max_ = 0.953	*k* = −9→9
3714 measured reflections	*l* = −16→8

### Refinement

**Table:**

Refinement on *F*^2^	Primary atom site location: structure-invariant direct methods
Least-squares matrix: full	Secondary atom site location: difference Fourier map
*R*[*F*^2^ > 2σ(*F*^2^)] = 0.053	Hydrogen site location: inferred from neighbouring sites
*wR*(*F*^2^) = 0.151	H-atom parameters constrained
*S* = 1.07	*w* = 1/[σ^2^(*F*_o_^2^) + (0.0613*P*)^2^ + 0.1694*P*] where *P* = (*F*_o_^2^ + 2*F*_c_^2^)/3
2575 reflections	(Δ/σ)_max_ < 0.001
181 parameters	Δρ_max_ = 0.20 e Å^−^^3^
1 restraint	Δρ_min_ = −0.28 e Å^−^^3^

### Special details

**Table:**

Geometry. All e.s.d.'s (except the e.s.d. in the dihedral angle between two l.s. planes) are estimated using the full covariance matrix. The cell e.s.d.'s are taken into account individually in the estimation of e.s.d.'s in distances, angles and torsion angles; correlations between e.s.d.'s in cell parameters are only used when they are defined by crystal symmetry. An approximate (isotropic) treatment of cell e.s.d.'s is used for estimating e.s.d.'s involving l.s. planes.
Refinement. Refinement of *F*^2^ against ALL reflections. The weighted *R*-factor *wR* and goodness of fit *S* are based on *F*^2^, conventional *R*-factors *R* are based on *F*, with *F* set to zero for negative *F*^2^. The threshold expression of *F*^2^ > σ(*F*^2^) is used only for calculating *R*-factors(gt) *etc*. and is not relevant to the choice of reflections for refinement. *R*-factors based on *F*^2^ are statistically about twice as large as those based on *F*, and *R*- factors based on ALL data will be even larger.

### Fractional atomic coordinates and isotropic or equivalent isotropic displacement parameters (Å^2^)

**Table:**

	*x*	*y*	*z*	*U*_iso_*/*U*_eq_	
C1	0.7607 (4)	0.1903 (4)	0.18562 (17)	0.0483 (6)	
H1A	0.7398	0.2592	0.2458	0.058*	
H1B	0.6839	0.1075	0.1981	0.058*	
C2	0.6871 (4)	0.3430 (3)	0.09029 (18)	0.0492 (6)	
H2A	0.5961	0.3147	0.0521	0.059*	
H2B	0.6093	0.4820	0.1083	0.059*	
C3	1.0544 (3)	0.1481 (3)	0.07401 (15)	0.0383 (5)	
C4	1.2831 (3)	0.0707 (3)	0.03509 (15)	0.0373 (5)	
C5	1.3404 (3)	0.1420 (3)	−0.06192 (15)	0.0414 (5)	
H5	1.2337	0.2373	−0.1034	0.050*	
C6	1.5554 (4)	0.0716 (3)	−0.09660 (15)	0.0416 (5)	
H6	1.5925	0.1195	−0.1615	0.050*	
C7	0.1900 (4)	0.5421 (5)	0.2448 (2)	0.0620 (8)	
C8	0.2163 (3)	0.3932 (3)	0.33993 (16)	0.0437 (5)	
C9	0.1925 (4)	0.2191 (4)	0.33805 (19)	0.0529 (6)	
H9	0.1691	0.1887	0.2765	0.064*	
C10	0.2027 (4)	0.0885 (4)	0.4264 (2)	0.0639 (7)	
H10	0.1849	−0.0275	0.4239	0.077*	
C11	0.2391 (4)	0.1307 (4)	0.5170 (2)	0.0620 (7)	
H11	0.2445	0.0452	0.5769	0.074*	
C12	0.2671 (3)	0.3009 (4)	0.51797 (16)	0.0507 (6)	
C13	0.2561 (3)	0.4345 (3)	0.43168 (17)	0.0462 (5)	
H13	0.2750	0.5497	0.4350	0.055*	
N1	0.8925 (3)	0.3081 (3)	0.03187 (14)	0.0482 (5)	
H1	0.9055	0.3822	−0.0226	0.058*	
N2	0.9901 (3)	0.0671 (3)	0.15833 (13)	0.0438 (5)	
H2	1.0721	−0.0439	0.1930	0.053*	
N3	0.3060 (4)	0.3471 (5)	0.61616 (18)	0.0776 (8)	
O1	0.1555 (3)	0.4959 (3)	0.16467 (14)	0.0884 (7)	
O2	0.2025 (3)	0.7013 (3)	0.25649 (18)	0.0916 (8)	
O3	0.3241 (4)	0.5037 (6)	0.6153 (2)	0.1100 (10)	
O4	0.3135 (4)	0.2288 (5)	0.69216 (16)	0.1205 (11)	

### Atomic displacement parameters (Å^2^)

**Table:**

	*U*^11^	*U*^22^	*U*^33^	*U*^12^	*U*^13^	*U*^23^
C1	0.0495 (13)	0.0543 (13)	0.0410 (12)	−0.0242 (11)	−0.0023 (10)	−0.0025 (10)
C2	0.0456 (13)	0.0468 (12)	0.0473 (13)	−0.0152 (10)	−0.0034 (10)	−0.0009 (10)
C3	0.0477 (12)	0.0387 (10)	0.0286 (10)	−0.0187 (9)	−0.0089 (9)	−0.0010 (8)
C4	0.0446 (12)	0.0356 (10)	0.0298 (10)	−0.0160 (9)	−0.0068 (8)	−0.0011 (8)
C5	0.0472 (12)	0.0380 (11)	0.0323 (11)	−0.0142 (9)	−0.0099 (9)	0.0053 (8)
C6	0.0516 (13)	0.0424 (11)	0.0273 (10)	−0.0197 (10)	−0.0045 (9)	0.0040 (8)
C7	0.0410 (13)	0.0717 (18)	0.0494 (16)	−0.0137 (12)	−0.0021 (11)	0.0233 (13)
C8	0.0359 (11)	0.0477 (12)	0.0367 (12)	−0.0129 (9)	−0.0031 (9)	0.0074 (9)
C9	0.0513 (14)	0.0599 (15)	0.0463 (14)	−0.0226 (11)	−0.0085 (11)	−0.0052 (11)
C10	0.0610 (16)	0.0510 (14)	0.080 (2)	−0.0292 (13)	−0.0097 (14)	0.0104 (13)
C11	0.0513 (15)	0.0686 (17)	0.0530 (16)	−0.0235 (13)	−0.0059 (12)	0.0225 (13)
C12	0.0350 (12)	0.0701 (16)	0.0336 (12)	−0.0131 (11)	−0.0033 (9)	−0.0013 (11)
C13	0.0362 (11)	0.0487 (12)	0.0484 (13)	−0.0153 (10)	−0.0014 (9)	−0.0037 (10)
N1	0.0481 (11)	0.0478 (10)	0.0372 (10)	−0.0146 (9)	−0.0047 (8)	0.0072 (8)
N2	0.0464 (10)	0.0434 (10)	0.0365 (10)	−0.0181 (8)	−0.0060 (8)	0.0062 (8)
N3	0.0438 (13)	0.124 (2)	0.0481 (14)	−0.0212 (14)	−0.0029 (10)	−0.0174 (15)
O1	0.0837 (14)	0.1023 (16)	0.0365 (10)	−0.0115 (12)	−0.0080 (9)	0.0177 (10)
O2	0.0766 (14)	0.0759 (14)	0.1167 (19)	−0.0418 (12)	−0.0346 (12)	0.0561 (13)
O3	0.0816 (17)	0.173 (3)	0.0934 (19)	−0.0593 (18)	0.0042 (13)	−0.0671 (19)
O4	0.0984 (18)	0.183 (3)	0.0363 (12)	−0.0305 (18)	−0.0134 (11)	0.0122 (15)

### Geometric parameters (Å, °)

**Table:**

C1—N2	1.462 (3)	C7—O2	1.257 (4)
C1—C2	1.531 (3)	C7—C8	1.514 (3)
C1—H1A	0.9700	C8—C9	1.380 (3)
C1—H1B	0.9700	C8—C13	1.387 (3)
C2—N1	1.468 (3)	C9—C10	1.388 (3)
C2—H2A	0.9700	C9—H9	0.9300
C2—H2B	0.9700	C10—C11	1.367 (4)
C3—N2	1.312 (3)	C10—H10	0.9300
C3—N1	1.317 (3)	C11—C12	1.366 (4)
C3—C4	1.476 (3)	C11—H11	0.9300
C4—C6^i^	1.393 (3)	C12—C13	1.380 (3)
C4—C5	1.394 (3)	C12—N3	1.481 (3)
C5—C6	1.383 (3)	C13—H13	0.9300
C5—H5	0.9300	N1—H1	0.8600
C6—C4^i^	1.393 (3)	N2—H2	0.8600
C6—H6	0.9300	N3—O4	1.221 (3)
C7—O1	1.246 (4)	N3—O3	1.222 (4)
			
N2—C1—C2	102.53 (17)	C9—C8—C13	118.9 (2)
N2—C1—H1A	111.3	C9—C8—C7	120.8 (2)
C2—C1—H1A	111.3	C13—C8—C7	120.2 (2)
N2—C1—H1B	111.3	C8—C9—C10	121.2 (2)
C2—C1—H1B	111.3	C8—C9—H9	119.4
H1A—C1—H1B	109.2	C10—C9—H9	119.4
N1—C2—C1	102.37 (17)	C11—C10—C9	119.9 (2)
N1—C2—H2A	111.3	C11—C10—H10	120.1
C1—C2—H2A	111.3	C9—C10—H10	120.1
N1—C2—H2B	111.3	C12—C11—C10	118.7 (2)
C1—C2—H2B	111.3	C12—C11—H11	120.7
H2A—C2—H2B	109.2	C10—C11—H11	120.7
N2—C3—N1	111.85 (19)	C11—C12—C13	122.8 (2)
N2—C3—C4	122.98 (18)	C11—C12—N3	118.7 (2)
N1—C3—C4	125.13 (18)	C13—C12—N3	118.4 (3)
C6^i^—C4—C5	119.30 (19)	C12—C13—C8	118.5 (2)
C6^i^—C4—C3	119.93 (18)	C12—C13—H13	120.7
C5—C4—C3	120.75 (18)	C8—C13—H13	120.7
C6—C5—C4	120.20 (19)	C3—N1—C2	110.95 (18)
C6—C5—H5	119.9	C3—N1—H1	124.5
C4—C5—H5	119.9	C2—N1—H1	124.5
C5—C6—C4^i^	120.50 (19)	C3—N2—C1	111.14 (17)
C5—C6—H6	119.7	C3—N2—H2	124.4
C4^i^—C6—H6	119.7	C1—N2—H2	124.4
O1—C7—O2	127.2 (2)	O4—N3—O3	124.9 (3)
O1—C7—C8	117.2 (3)	O4—N3—C12	117.8 (3)
O2—C7—C8	115.6 (3)	O3—N3—C12	117.3 (3)

Symmetry codes: (i) −*x*+3, −*y*, −*z*.

### Hydrogen-bond geometry (Å, °)

**Table:**

*D*—H···*A*	*D*—H	H···*A*	*D*···*A*	*D*—H···*A*
C6—H6···O2^ii^	0.93	2.48	3.283 (3)	144
N1—H1···O1^iii^	0.86	1.97	2.763 (3)	153
N2—H2···O2^iv^	0.86	1.80	2.646 (3)	166

Symmetry codes: (ii) −*x*+2, −*y*+1, −*z*; (iii) −*x*+1, −*y*+1, −*z*; (iv) *x*+1, *y*−1, *z*.
